# Pre-post effects of a tetanus care protocol implementation in a sub-Saharan African intensive care unit

**DOI:** 10.1371/journal.pntd.0006667

**Published:** 2018-08-30

**Authors:** Riaz Aziz, Soledad Colombe, Gibonce Mwakisambwe, Solomon Ndezi, Jim Todd, Samuel Kalluvya, Halinder S. Mangat, Reed Magleby, Arndt Koebler, Bernard Kenemo, Robert N. Peck, Jennifer A. Downs

**Affiliations:** 1 Intensive Care Department, Bugando Medical Centre, Mwanza, Tanzania; 2 Center for Global Health, Department of Medicine, Weill Cornell Medicine, New York, NY, United States of America; 3 Department of Applied Biostatistics, London School of Hygiene and Tropical Medicine, London, United Kingdom; 4 Department of Medicine, Bugando Medical Centre, Mwanza, Tanzania; 5 Department of Neurology, New York-Presbyterian Hospital/Weill Cornell Medical Centre, New York, NY, United States of America; 6 Medical Mission Institute, Wurzburg, Germany; University of California San Diego School of Medicine, UNITED STATES

## Abstract

**Background:**

Tetanus is a vaccine-preventable, neglected disease that is life threatening if acquired and occurs most frequently in regions where vaccination coverage is incomplete. Challenges in vaccination coverage contribute to the occurrence of non-neonatal tetanus in sub-Saharan countries, with high case fatality rates. The current WHO recommendations for the management of tetanus include close patient monitoring, administration of immune globulin, sedation, analgesia, wound hygiene and airway support [1]. In response to these recommendations, our tertiary referral hospital in Tanzania implemented a standardized clinical protocol for care of patients with tetanus in 2006 and a subsequent modification in 2012. In this study we aimed to assess the impact of the protocol on clinical care of tetanus patients and their outcomes.

**Methods and findings:**

We examined provision of care and outcomes among all patients admitted with non-neonatal tetanus to the ICU at Bugando Medical Centre between 2001 and 2016 in this retrospective cohort study. We compared three groups: the pre-protocol group (2001–2005), the Early protocol group (2006–2011), and the Late protocol group (2012–2016) and determined associations with mortality by univariable logistic regression.

We observed a significant increase in provision of care as per protocol between the Early and Late groups. Patients in the Late group had a significantly higher utilization of mechanical ventilation (69.9% vs 22.0%, p< 0.0001), provision of surgical wound care (39.8% vs 20.3%, p = 0.011), and performance of tracheostomies (36.8% vs 6.7%, <0.0001) than patients in the Early group. Despite the increased provision of care, we found no significant decrease in overall mortality in the Early versus the Late groups (55.4% versus 40.3%, p = 0.069), or between the pre-protocol and post-protocol groups (60.7% versus 50.0%, p = 0.28). There was also no difference in 7-day ICU mortality (30.1% versus 27.8%, p = 0.70). Analysis of the causes of death revealed a decrease in deaths related to airway compromise (30.0% to 1.8%, p<0.001) but an increase in deaths due to presumed sepsis (15.0% to 44.6%, p = 0.018).

**Conclusion:**

The overall mortality in patients suffering non-neonatal tetanus is high (>40%). Institution of a standardized tetanus management protocol, in accordance with WHO recommendations, decreased immediate mortality related to primary causes of death after tetanus. However, this was offset by an increase in death due to later ICU complications such as sepsis. Our results illustrate the complexity in achieving mortality reduction even in illnesses thought to require few critical care interventions. Improving basic ICU care and strengthening vaccination programs to prevent tetanus altogether are essential components of efforts to decrease the mortality caused by this lethal, neglected disease.

## Author summary

Tetanus is a disease characterized by violent, repetitive muscular spasms that are frequently lethal. It rarely occurs in high-income countries due to a highly effective vaccine coupled with a strong immunisation system. Tanzania is a large low-income country in East Africa. Its size and rapidly growing population are straining factors for the immunisation programme. Women typically receive tetanus booster vaccinations during antenatal care, while men remain at risk for tetanus as there is no additional system in place to ensure post-infancy vaccination. In 2006, our hospital in northwest Tanzania implemented a standardized management protocol for the frequent cases of tetanus that we admit. We assessed the impact of this protocol on the care and survival of these patients, most of whom are between the ages of 20 and 50 years. Although we observed improving clinical care over a 16-year period, this increased care did not result in the reduction in mortality that had been expected. Our work reflects the complexity of this neglected disease, the challenges associated with protocolised care provision in such settings, and the importance of monitoring the effects of interventions following their implementation in low-income countries. Given the devastating personal and economic consequences of tetanus for patients and their families, we also highlight the urgent need to ensure immunisation for these vulnerable men who are the economic backbone for households and the country.

## Introduction

Despite being a vaccine-preventable disease, tetanus is frequently encountered in sub-Saharan Africa [2,3]. The incidence of non-neonatal tetanus cases has fallen since the initiation of the vaccination programme but the number of cases remains high, with 4,604 non-neonatal cases reported in 2016 in the African region^,^[4] and likely many more that were not reported [5]. Among global tetanus deaths, 44% occur in sub-Saharan Africa and the highest proportion of these is in East Africa [6].

Inadequate vaccination is cited as the primary causative factor for tetanus despite the availability of a highly effective vaccine [7,8]. In Tanzania, national tetanus vaccine coverage is 87% in children less than 1 year, as determined by history and vaccination cards, but regional discrepancies are high [9]. In Mwanza, where our hospital is located, only 70% of infants received all basic vaccines in 2015 [10]. In addition, because the Tanzanian vaccination programme focuses on children under the age of 1 and pregnant women who attend antenatal clinic, there is currently no system in place to ensure booster vaccination for men past the infancy doses, despite recommendations by the WHO [11]. This would explain why young men are the most at risk of tetanus infection in Tanzania and many other sub-Saharan African countries [3]. A recent study from Tanzania showed that only 28% of men older than 15 years were seroprotected against tetanus [12]. This has tremendous economic and social consequences: in 2015 the WHO calculated the cost of a single dose of tetanus vaccine at $0.14 [13] whereas the cost of caring for patients with tetanus in low- and middle-income countries ranges from $78 [14] to $900 [15]. In Tanzania, 55% of the population lives in extreme poverty (less than $1.25 per day) and men are the main financial providers, so the socioeconomic impact is tremendous.

Lack of medications, inadequate implementation of proven treatment interventions, high treatment cost for patients, and long distance to specialised centres have been cited as additional key reasons for high tetanus-associated mortality in sub-Saharan Africa [3,16,17]. Studies of targeted approaches to address these barriers are lacking. Therefore, our goal was to conduct a quality-improvement project to assess whether the utilization of a standardized hospital protocol for management of tetanus was effective in reducing mortality. Our study was possible because in 2006 our Tanzanian referral hospital implemented a hospital protocol to be used for management of all patients admitted to our ICU with tetanus, with further updates in 2012. We sought to determine whether the implementation of this protocol had any impact on provision of clinical care and patient outcomes. We hypothesised that protocol-driven implementation of proven tetanus interventions would increase over the study period, and that tetanus mortality would decrease.

## Methods

### Ethics statement

Ethical approval for the conduct of this study was obtained from the joint Catholic University of Health and Allied Sciences (CUHAS)/BMC Research Ethics and Review Committee (BREC/001/18/2008), the National Institute for Medical Research (NIMR/HQ/R.8c/Vol. IX/1085), and Weill Cornell Medicine (1108010827).

### Study setting

We conducted a retrospective cohort study of all patients who had a diagnosis of tetanus and were admitted to the Intensive Care Unit (ICU) of Bugando Medical Centre (BMC), Tanzania, from May 2001 to September 2016. BMC is a public tertiary referral hospital located in Mwanza, a north-western city on the shores of Lake Victoria. Mwanza is the second largest city in Tanzania and BMC serves the 15 million people of the Lake Zone.

The ICU admits approximately 500 patients annually from all disciplines, with specialists from internal medicine and anaesthesia providing the majority of the ICU care. The ICU has 13 beds with 7 mechanical ventilators, pressurised wall oxygen, suction, and bedside monitors. The monitors display non-invasive blood pressures, saturations and electrocardiography. There is regular availability of intravenous (IV) fluids, antibiotics, adrenaline, and dopamine. Central lines and noradrenaline are intermittently available. The nurse to patient ratio is 1:2, with most of the nurses having no formal training in intensive care medicine.

**Fig 1:**
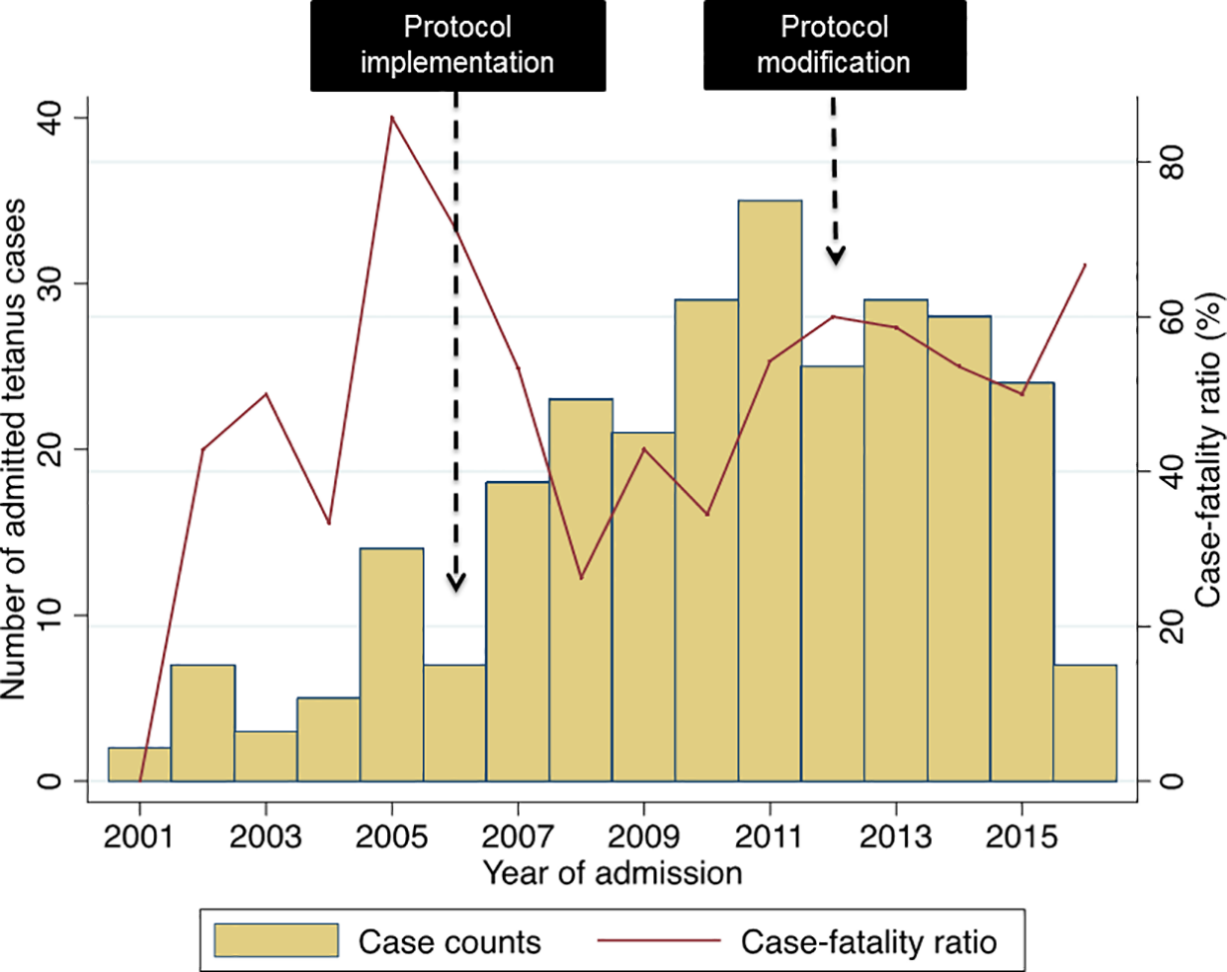
Case-fatality ratio among tetanus patients by year of admission over the period of 2001–2016.

In view of high mortality rates and in an effort to improve the quality of care for patients, BMC implemented a hospital protocol to optimize care of tetanus patients in 2006, in accordance with WHO recommendations. The hospital protocol can be seen in the supporting information (Supporting information (S1); Fig 1. Bugando Medical Centre tetanus management protocol) In 2012, several additional key interventions were implemented in the ICU, including employment of a dedicated ICU physician and an agreement with the surgical department that tracheostomies would be performed as soon as possible for tetanus patients admitted to the ICU.

The stepwise protocol is organised based on clinical urgency of interventions. The initial stage focuses on airway management either in the form of oral intubation or immediate tracheostomy. There was no specific criterion or threshold for initiation of mechanical ventilation during the entire study period and this decision was made at the discretion of the on-duty physician. This is followed by emphasis on early prescription and administration of immune globulin, antibiotics, spasm control with magnesium sulphate and benzodiazepines with accompanying analgesia, fluid management to avoid acute kidney injury and deep vein thrombosis prophylaxis. Wound care is also included in the early stages with surgical consultation for wound debridement if needed. Farmers and manual workers were identified, from previous work, as the key at-risk group, and most present with identifiable wounds.

The next stage focuses on monitoring the patient’s response to interventions and conducting investigations. Instructions are provided on how to adjust prescribed medication to get the desired outcomes. The final stage focuses on the recovery phase of care with instructions of down-titrating the medications and ensuring immunisation prior to ICU discharge.

### Study procedures

According to our hospital protocol, all patients presenting to the hospital with tetanus are admitted directly to the ICU. We identified patients with non-neonatal tetanus using either the ICU admission registry or the separate inpatient medical registry. Patients were diagnosed based on clinical findings of rigidity and/or spasms often preceded by a penetrating injury. Medical notes were sought from the medical records department. Detailed information on care was only available for patients after 2008. The main sources of information were the ICU admission notes, ward round notes and daily charts. We collected data on age, sex, place of residence, time taken to present to hospital, provision of clinical care, time to care provision, and outcomes.

### Data analysis

We categorized patients into 3 groups based on the year of presentation. The pre-protocol group included patients admitted with tetanus prior to the protocol implementation in 2006 (2001–2006). The Early group included patients admitted between 2006 and 2011, and the Late group included patients admitted between 2012 (the year in which the hospital tetanus protocol was modified) and 2016.

The primary study outcomes were care provision for patients, 7-day mortality in the ICU, and overall mortality. The specific care interventions that we examined included administration of immune globulin (early in the emergency department or late in the ICU), surgical wound care, administration of antibiotics, and airway management in the form of mechanical ventilation and tracheostomy placement.

Statistical analysis was performed using STATA 14.0 (College Station, Texas, USA) and all data was anonymized during the analysis. Descriptive analysis of baseline variables was performed to summarize patient characteristics. Categorical variables were described using proportions and continuous variables were described using medians and interquartile ranges. We compared overall mortality between the pre- and post-protocol groups. We explored the differences between the Early and Late groups by chi-squared test for categorical variables and Wilcoxon rank-sum test for continuous variables. Overall and 7-day case-fatality rates were calculated and compared between the groups. We assessed potential factors associated with mortality by univariable and multivariable logistic regression for the Early and Late groups. All statistical tests were performed at a 5% significance level.

## Results

A total of 277 patients were admitted to the BMC ICU with tetanus between May 2001 and September 2016. No cases of neonatal tetanus were admitted; all patients were 8 years older and above. Thirty-one (11.2%) patients were admitted before January 2006 and were classified in the pre-protocol group, 133(48.0%) patients were classified in the Early group, and 113 (40.8%) were classified in the Late group. Medical files were available for 162 patients (59 in the Early group and 103 in the Late group).

When comparing the pre and post-protocol groups, both were mostly male (12/14 (85.7%) and 211/246 (85.8%) respectively, p = 0.995), and there was no significant difference in age (30.0 [21–42] years and 30.0 [19–46] years respectively, p = 0.86, **Table 1**). Overall case fatality was similar in both the pre- and post-protocol group (17/28 (60.7%) and 119/238 (50.0%), p = 0.28). The case-fatality rate by year of admission is presented in **Fig 1**.

**Table 1:** Baseline demographic characteristics for the early and late groups.

Variable	Early group 2006–2011	Late group 2012–2016	p-value*
	n/N (%) or median (IQR)	n/N (%) or median (IQR)	
**Demographic characteristics**			
**Sex (Male)**	110/133 (82.7%)	101/113 (89.4%)	0.13
**Age at presentation**			
Overall	31.0 [20.0–44.0]	30.0 [18.5–52.0]	0.71
Male	30.5 [22.0–41.5]	32.0 [19.5.-54.0]	0.19
Female	47.0 [15.0–68.0]	16.0 [12.0–29.5]	0.15
**Distance travelled (kilometres)**	55.0 [5.9–154.9]	62.0 [5.9–154.9]	0.84
**Presentation to hospital**			
**Time from symptoms to presentation (days)**	3 [1–7]	3 [1–4]	**0.042**
	0–2 days: 18 (30.5%)	0–2 days: 49 (47.6%)	
	3–4 days: 19 (32.2%)	3–4 days: 35 (34.0%)	
	5–6 days: 4 (6.8%)	5–6 days: 3 (2.9%)	
	7+ days: 18 (30.5%)	7+ days: 16 (15.5%)	
**Symptoms**			
Presence of fever	4/59 (6.8%)	14/103 (13.6%)	0.21
Generalized clinical presentation (versus local)	59/59 (100.0%)	98/103 (95.2%)	0.16
Location of wound			
*Leg*	14/59 (23.7%)	32/103 (31.1%)	0.37
*Foot*	19/59 (32.2%)	32/103 (31.1%)	1.00
*Hands*	9/59 (15.3%)	13/103 (12.6%)	0.64
*Chest*	0/59 (0.0%)	2/103 (1.9%)	0.53
*Circumcision*	2/59 (3.4%)	4/103 (3.9%)	1.00
*Scrotum*	0/59 (0.0%)	1/103 (1.0%)	1.00
*Head/face*	6/59 (10.2%)	9/103 (8.7%)	0.78
*Arm*	3/59 (5.1%)	3/103 (2.9%)	0.67
*Back*	4/59 (6.8%)	0/103 (0.0%)	0.016
*Axilla*	0/59 (0.0%)	2/103 (1.9%)	0.53
*Abortion*	0/59 (0.0%)	1/103 (1.0%)	1.00
*Unknown*	2/59 (3.4%)	4/103 (3.9%)	1.00

*p-value for the overall difference between the two groups calculated by Chi-square test or Fisher’s Exact test for categorical variables and Wilcoxon rank-sum test for continuous variables.

When comparing the Early and Late groups, for whom detailed medical record data was available, we found no differences between the two groups with respect to sex, age, or distance travelled to reach BMC (**Table 1**). The groups had similar median time intervals from the onset of symptoms to hospital presentation, though significantly more people in the Early group presented later (3 [1–7] versus 3 [1–4] days, p = 0.042). The Early group also experienced significantly more wounds to the back than the Late group. No other significant differences in clinical presentation were observed.

Care interventions significantly differed between the two post-protocol groups (**Table 2**). We observed significant increases in surgical wound care, initiation of mechanical ventilation and performance of tracheostomies in the Late group compared to the Early group. Both groups had similar in-hospital rates of immune globulin administration, but 57/95 (60.0%) of patients in the Late group received early administration of immune globulin in the emergency department compared to 9/50 (18.0%) of patients in the Early group (p<0.001). We additionally observed a reduction in the time taken to initiating mechanical ventilation (2.0 [2.0–4.0] days in the Early group versus 0.0 [0.0–2.0] in the Late group, p = 0.0030) and in performing tracheotomies in those for whom mechanical ventilation was initiated (12.0 [8.5–18.5] days in the Early group versus 1.0 [0.0–8.0] in the Late group, p = 0.023). All patients in the Early and Late group received antibiotics.

**Table 2:** Care interventions done as per protocol for the early (2006–2011) and the late group (2012–2016)*.

Variable	Early group 2006–2011	Late group 2012–2016	p-value**
	n/N (%) or median (SD)	n/N (%) or median (SD)	
**Care interventions**			
**Received immune globulin (IG)**	50/59 (84.7%)	95/102 (93.1%)	0.086
**Timing of IG administration**			**<0.001**
Emergency room (Early)	9/50 (18.0%)	57/95 (60.0%)	
ICU (Late)	41/50 (82.0%)	38/95 (40.0%)	
**Received surgical intervention**	12/59 (20.3%)	41/100 (41.0%)	**0.008**
**Type of surgical intervention**			1.00
Debridement	12/12 (100.0%)	40/41 (97.6%)	
Amputation	0/12 (0.0%)	1/41 (2.4%)	
**Received mechanical ventilation**	13/59 (22.0%)	72/103 (69.9%)	**<0.001**
**Received tracheostomy**	4/59 (6.8%)	38/97 (39.2%)	**<0.001**
**Timing of interventions (in days)**			
**Time to surgical intervention**	1.5 [1.0–3.0]	2.0 [1.0–3.0]	0.96
**Time to mechanical ventilation**	2.0 [2.0–4.0]	0.0 [0.0–2.0]	**0.0030**
**Time from initiation of mechanical ventilation to tracheostomy**	12.0 [8.5–18.5]	1.0 [0.0–8.0]	**0.023**
**Length of stay in the ICU**	9.0 [2.5–22.0]	12.0 [5.0–22.0]	0.12
**Length of stay at BMC**	22.0 [6.0–31.0]	14.0 [6.0–31.0]	0.38

*Only patients with charts available for review were included in this analysis.

**p-value for the overall difference between the two groups calculated by Chi-squared or Fisher’s Exact test for categorical variables and Wilcoxon rank-sum test for continuous variables.

Contrary to our hypothesis, we did not find a significant reduction in the overall hospital case-fatality ratios between the Early and the Late groups. In contrast, there was a trend towards an increase in mortality from 56/126(44.4%) in the Early group to 63/112 (56.3%) in the Late group (p = 0.069). Additionally, there was no change in 7-day case-fatality ratio (43/126 (34.1%) versus 35/112 (31.3%), p = 0.64). The Early group had 6/20 (30.0%) deaths attributed to loss of airway, mostly due to laryngospasm, whereas in the Late group, this accounted for only 1/56 (1.8%) death (p = 0.001). Sepsis accounted for 3/20 (15.0%) of all deaths in the Early group and 25/56 (44.6%) in the Late group (p = 0.029) (**Table 3**). Of note, there were no other clear changes between the Early and Late groups such as ICU staff: patient ratios, nurse training, ICU admission numbers, timeliness of antibiotic administration, or decontamination procedures.

**Table 3:** Overall ICU mortality, 7-day mortality and causes of death in the early and the late groups.

	Early group 2008–2011	Late group 2012–2016	p-value*
	n/N(%)	n/N(%)	
Overall ICU mortality	56/126 (44.4%)	63/112 (56.3%)	0.069
7 day ICU mortality	43/126 (34.1%)	35/112(31.3%)	0.64
**Cause of death**			
Sepsis	3/20 (15.0%)	25/56 (44.6%)	**0.029**
Airway obstruction	6/20 (30.0%)	1/56 (1.8%)	**0.001**
Respiratory failure	6/20 (30.0%)	11/56 (19.6%)	0.36
Autonomic failure	5/20 (25.0%)	14/56 (25.0%)	1.00

*p-value for the overall difference between the two groups calculated by Chi-square test or Fisher’s exact test as appropriate.

All variables listed in Table 1 were analysed as possible predictors of overall ICU mortality in the Early and Late groups and are presented in **Table 4**. In the Early group, patients with longer time from symptom onset to presentation at the hospital had lower odds of death compared to patients with shorter time to presentation (OR = 0.68 [0.52–0.90] for each day delay in presenting for care, p = 0.007). Patients for whom mechanical ventilation was initiated had higher odds of death compared to patients not receiving mechanical ventilation (OR = 4.53 [1.24–16.58], p = 0.022). The increased odds of death may in part have been related to development of sepsis, though numbers were too small to draw conclusions. In the Late group, 25 out of 72 (34.7%) patients who were mechanically ventilated developed sepsis, compared with 3 out of 13 (23.1%) in the Early group (p = 0.53).

**Table 4:** Results of univariable analysis of predictors of overall mortality in the early and late groups.

Variable	Early Group Odds ratio [95%CI]	P-value	Late Group Odds ratio [95%CI]	P-value
**Demographic Information**				
Sex (male)	0.49 [0.19–1.24]	0.13	1.93 [0.57–6.51]	0.29
Age (years)	1.02 [0.997–1.037]	0.10	1.05 [1.02–1.07]	**<0.001**
Distance travelled to BMC (Kilometres)	0.99 [0.99–1.00]	0.16	1.00 [0.99–1.00]	0.94
**Care Interventions**				
Administration of Immune globulin	0.34 [0.08–1.46]	0.15	0.46 [0.09–2.51]	0.37
Late administration of Immune Globulin (compared to early)	1.63 [0.30–8.93]	0.58	2.29 [0.98–5.36]	0.055
Surgical intervention	0.59 [0.14–2.47]	0.47	0.61 [0.27–1.36]	0.23
Mechanical Ventilation	4.53 [1.24–16.58]	**0.022**	1.41 [0.61–3.28]	0.43
**Tracheotomy**	0.63 [0.061–6.49]	0.70	0.91[0.40–2.07]	0.82
**Timing of interventions (odds ratio for each increasing day)**				
Time from symptom onset to attending care	0.68 [0.52–0.90]	**0.007**	0.99 [0.91–1.07]	0.80
Time from presentation to surgical intervention	1.04 [0.91–1.20]	0.55	0.94 [0.79–1.11]	0.45
Time from presentation to initiation of mechanical ventilation	0.71 [0.40–1.26]	0.24	1.63 [1.06–2.52]	**0.028**
Time from presentation to tracheotomy	—**	—	0.95 [0.88–1.04]	0.27

**—Only one data point for timing of tracheotomy for a patient who died.

In the Late group, older patients as well as patients with longer time from presentation to initiation of mechanical ventilation had higher odds of death (OR = 1.05 [1.02–1.07] for each increasing year of age, p<0.001 and OR = 1.63 [1.06–2.52] for each day delay in receiving mechanical ventilation, p = 0.028 respectively). Multivariable analysis showed that the factors associated with mortality in the Early group were age (OR = 1.04 [95% CI 1.00–1.08], P = 0.03), mechanical ventilation (OR = 26.7 [95% CI 2.06–274.07], p = 0.006) and time from symptom onset to attending care (OR = 0.50 [95% CI 0.32–0.81), p = 0.006). In the Late group, the factors that remained significantly associated with mortality were age (OR = 1.03[95% CI 1.00–1.06], p = 0.01) and time from presentation to initiation of mechanical ventilation (OR = 1.74[95% CI 1.07–2.81], p = .024).

## Discussion

Our work demonstrates that the use of a standardized protocol was associated with a significant improvement in the implementation of specific interventions that have been shown to reduce mortality from tetanus. However, this led to an increase in invasive procedures, and therefore the anticipated reduction in overall mortality over a 10-year period was not seen. While the deaths due to respiratory failure and airway obstruction decreased, those due to sepsis increased. This ultimately led to longer ICU stays over the 10-year period without improving mortality. To our knowledge, this is the first study from sub-Saharan Africa to look at the impact of protocolised ICU care for tetanus patients. Our findings suggest the urgent need for additional work to optimize ICU care to help offset the mortality from secondary causes which arise due to gains from improvement in immediate survival. Furthermore, data from our study shows mortality from tetanus remains high and that efforts to improve adult immunization to prevent this neglected disease should be prioritized.

Most previous studies have highlighted poor provision of clinical care as a key contributory factor to observed high mortalities. In the Late period of our protocol implementation, patients experienced higher levels of clinical care as compared to other studies. Rates of mechanical ventilation (69.9% in our study vs 10.5% in other studies) [16,17], administration of immune globulin (93.1% vs 9–65%) [17,18], surgical wound care (41.0% vs 11.8%) [18], administration of antibiotics (100% vs 58%) [17], and tracheotomies (39.2% vs 11%) [16] were all higher in the Late period of our study compared to rates reported in other studies. A structured approach via a standardized protocol was likely a key contributory factor to the increase in the clinical care. In spite of the protocol, the mortality rates we have reported in both time periods of our study are very similar to rates reported in other studies from similar settings.

There is mixed evidence for the effectiveness of protocol standardization for medical care both globally [19,20] and also in low- and middle-income countries [21,22]. Nonetheless, protocolised care is still common, especially in environments where specialised care is not readily available and where providers’ levels of training and expertise may be variable. Despite highly significant increases in clinical care provision with earlier administration of immune globulin, an increase in surgical wound care, and an increase in mechanical ventilation and tracheostomies, our results showed no improvement in mortality. In fact, we even observed a non-significant trend towards increased mortality in the Late years of implementation of the protocol compared to the Early years (56.3% versus 44.4%, p = 0.069). The similarity in demographics between the two groups reinforces the hypothesis that the differences in mortality may be due to the protocol itself.

Implementation of the hospital protocol for tetanus management appears to have led to a shift in the causes of death among tetanus patients. In the Early years after protocol implementation, airway obstruction and respiratory failure were the most frequent causes of death, consistent with findings from other studies [3,16]. In contrast, sepsis was the most likely cause of death in the Late group, increasing from 15% at baseline to 45% in the Late group. It is likely that increased rates of mechanical ventilation and performance of tracheostomies contributed to a decline in the number of airway/respiratory deaths but that this overall increase in interventions led to an increase in the number of deaths by sepsis, for at least three reasons. First, the Late group underwent more invasive procedures including mechanical ventilation, surgical tracheostomies and surgical wound treatment, each of which increases the risk of hospital-acquired infections (HAIs). Second, our clinical experience suggests that patients in the Late group also more frequently had central venous catheters inserted, which poses an additional risk factor for HAIs. Third, patients in the Late group spent more days in the ICU, often immobilized and at increased risk for other causes of in-hospital mortality such as pulmonary embolism, urosepsis and bedsores. Of note, at present there is no specific aspect of the protocol that focuses on reducing HAIs or other critical illness-associated morbidity.

There are approximately 27 studies involving more than 25,000 patients with tetanus over a 60-year period issued from the African continent [3]. Most of the reported studies have been done at tertiary level health facilities, similar to our hospital. The median age, male predominance, median time to presentation, clinical presentation, and hospital lengths of stay are similar between our two groups and also similar to other studies of tetanus patients in Africa [3]. Most of the studies reported similar high overall hospital fatalities [3]. All of this suggests that our hospital setting is similar to many others in sub-Saharan Africa and that our finding that tetanus protocol implementation did not improve mortality is likely to be generalizable as well.

Our results are to be interpreted in light of some limitations. First, due to our inability to locate medical records for some of the tetanus patients, we were not able to document the care provided in all cases but only to document basic demographic characteristics and outcomes. Furthermore, additional data that would have been informative, such as trends in antibiotic resistance over time and the specific antibiotics administered, were not available. These limitations highlight the complexity of implementing a hospital protocol and the urgent need for additional studies in this area.

In summary, we have demonstrated a significant increase in clinical care in accordance with a standardized protocol for the treatment of tetanus patients. The protocol has not led to the anticipated reduction in patient mortality. The unchanged mortality rate, with a shift in causes of death, highlights several key points for consideration when protocols are implemented in resource-limited settings. First, implementation of protocolised care in resource-limited settings is highly complex and requires in-depth monitoring and assessment of patients, staff, and procedures. In our hospital, we are now working to implement infection control policies and determine antibiotic resistance patterns in an effort to decrease HAIs. We will continue to monitor the effect of this intervention and to consider other possible interventions to decrease the mortality of tetanus patients. In addition, management and early recognition of sepsis is extremely complex in resource-limited settings and more surveillance is needed. Finally, we strongly call for an increase in vaccination coverage for at-risk men in sub-Saharan Africa, beginning with the highest-risk groups such as farmers and motorcyclists [5,23], with the aim of eliminating this preventable, lethal disease.

## Supporting information

S1 Checklist STROBE checklist. (DOC)

S1 Fig (PDF)

## References

[1] World Health Organization (WHO). WHO position paper: Tetanus Vaccine [Internet]. 2004 pp. 25–40.

[2] World Health Organization (WHO). Tetanus [Internet]. 2017 [cited 16 Jan 2018]. Available: http://www.who.int/immunization/diseases/tetanus/en/

[3] Woldeamanuel YW, Andemeskel AT, Kyei K, Woldeamanuel MW, Woldeamanuel W. Case fatality of adult tetanus in Africa: Systematic review and meta-analysis. J Neurol Sci. 2017;368: 292–299. 10.1016/j.jns.2016.07.025 27538652

[4] World Health Organization (WHO). Global and regional immunization profile—Africa Region [Internet]. 2017 [cited 16 Jan 2018]. Available: http://www.who.int/immunization/monitoring_surveillance/data/gs_afrprofile.pdf?ua=1

[5] Aziz R, Peck RN, Kalluvya S, Kenemo B, Chandika A, Downs JA. Tetanus in adult males, Bugando Medical Centre, United Republic of Tanzania. Bull World Heal Organ. 2017;95: 779–784. 10.2471/BLT.16.185546 PMC5677607 29147059

[6] Kyu HH, Mumford JE, Stanaway JD, Barber RM, Hancock JR, Vos T, et al Mortality from tetanus between 1990 and 2015: findings from the global burden of disease study 2015. BMC Pub Heal. BMC Public Health; 2017; 1–17. 10.1186/s12889-017-4111-4 28178973 PMC5299674

[7] Centers for Disease Control and Prevention. Tetanus In: Hamborsky J, Kroger A, Wolfe S, editors. Epidemiology and Prevention of Vaccine-Preventable Diseases. 13th ed Washington, DC: Public Health Foundation; 2015 Available: https://www.cdc.gov/vaccines/pubs/pinkbook/tetanus.html

[8] Centers for Disease Control and Prevention. CDC Tetanus Surveillance [Internet]. 2017. Available: http://www.cdc.gov/vaccines/

[9] Ministry of Health and Social Welfare—Tanzania mainland. Expanded Programme on Immunisation: 2010–2015 comprehensive multi year plan. 2015; Available: www.gavi.org/country/tanzania/…/comprehensive-multi-year-plan-for-2010-2015/

[10] Ministry of Health Community Development Gender Elderly and Children (MoHCDGEC); National Bureau of Statistics; Office of the Chief Government Statistician; and ICF. 2015–16 TDHS-MIS Key Findings. [Internet]. Rockville, MD; 2016. Available: https://www.dhsprogram.com/pubs/pdf/SR233/SR233.pdf

[11] States M, Strategic WHO, Group A, Grade T, Sage T. Weekly epidemiological record Relevé épidémiologique hebdomadaire. 2017; 53–76.

[12] Scobie HM, Patel M, Martin D, Mkocha H, Njenga SM, Odiere MR, et al Tetanus Immunity Gaps in Children 5–14 Years and Men &gt; = 15 Years of Age Revealed by Integrated Disease Serosurveillance in Kenya, Tanzania, and Mozambique. Am J Trop Med Hyg. 2016;96: 415–420. 10.4269/ajtmh.16-0452 27920395 PMC5303047

[13] Wolfson LJ, Gasse F, Lee-Martin SP, Lydon P, Magan A, Tibouti A, et al Estimating the costs of achieving the WHO-UNICEF Global Immunization Vision and Strategy, 2006–2015. Bull World Heal Organ. 2008;86: 27–39. 10.2471/BLT.07.045096 18235887 PMC2647343

[14] Habte-Gabr E., Mengistu M. Tetanus in Gondar Public Health College Hospital, Ethiopia: A review of 72 cases. Eth Med J. 1978;16: 53–61. 720326

[15] Miranda-Filho DB, Ximenes RAA, Siqueira-Filha NT, Santos AC. Incremental costs of treating tetanus with intrathecal antitetanus immunoglobulin. Trop Med Int Heal. 2013;18: 555–563. 10.1111/tmi.12091 23461581

[16] Amare A, Yami A. Case-fatality of adult Tetanus at Jimma University Teaching Hospital, Southwest Ethiopia. Afr Heal Sci. 2011;11: 36–40. PMC3092314 21572855

[17] Hesse I, Mensah A. Adult tetanus in Accra, Why the high mortality? An audit of clinical managment of tetanus. WAJM. 2005;24: 157–161. 10.4314/wajm.v24i2.28188 16092319

[18] Derbie A, Amdu A, Alamneh A, Tadege A, Solomon A, Elfu B, et al Clinical profile of tetanus patients attended at Felege Hiwot Referral Hospital, Northwest Ethiopia: a retrospective cross sectional study. Springerplus. 2016;5: 892 10.1186/s40064-016-2592-8 27386340 PMC4923016

[19] Mouncey PR, Osborn TM, Power GS, Harrison DA, Sadique MZ, Grieve RD, et al Protocolised Management In Sepsis (ProMISe):A multicentre randomised controlled trial of the clinical effectiveness and cost-effectiveness of early, goal-directed, protocolised resuscitation for emerging septic shock. Heal Technol Assess. 2015;19: 1–150. 10.3310/hta19970 26597979 PMC4781482

[20] Blackwood B, Alderdice F, Burns K, Cardwell C, Lavery G, O’Halloran P. Use of weaning protocols for reducing duration of mechanical ventilation in critically ill adult patients: Cochrane systematic review and meta-analysis. BMJ. 2011;342: c7237–c7237. 10.1136/bmj.c7237 21233157 PMC3020589

[21] Jacob ST, Banura P, Baeten JM, Moore CC, Meya D, Nakiyingi L, et al The impact of early monitored management on survival in hospitalized adult Ugandan patients with severe sepsis: a prospective intervention study. Crit Care Med. 2013;40: 2050–2058. 10.1097/CCM.0b013e31824e65d7 PMC3378757 22564958

[22] Andrews B, Muchemwa L, Kelly P, Lakhi S, Heimburger DC, Bernard GR. Simplified Severe Sepsis Protocol. Crit Care Med. 2014;42: 2315–2324. 10.1097/CCM.0000000000000541 25072757 PMC4199893

[23] Bankole I Danesi M. Characteristics and outcome of tetanus in adolescent and adult patients admitted to the Lagos University Teaching Hospital between 2000 and 2009. J Neurol Sci. 2012;323: 201–204. 10.1016/j.jns.2012.09.017 23069727

