# Supplementary material for: Pre-post effects of a tetanus care protocol implementation in a sub-Saharan African intensive care unit
Source: PLoS Negl Trop Dis. 2018 Aug 30;12(8):e0006667. doi: 10.1371/journal.pntd.0006667 (PMC6116918; doi:10.1371/journal.pntd.0006667)
Supplement: S1 Fig — (PDF) [file pntd.0006667.s002.pdf]

Tetanus

Patient with clinical symptoms of tetanus

→

Admit to ICU

Initial Prescriptions

- ATS 10,000 IU stat
- 1st dose before antibiotics or debridement
- Metronidazole 500mg i.v. TDS
- $MgS0_4$ 5g IV STAT (slowly) (or 75mg/kg)
- $MgS0_4$ 2.5g IV 2 hourly
- Diazepam 20mg IV TDS
- Midazolam 5mg IV PRN for break through spasms
- DVT / stress ulcer prophylaxis
- IV fluids (see below)

**use weight-based dosing in children <15 years old*

Consider

- If patient has severe spasms:
  →Phenobarbitone 120mg IV BD
- If RBG is > 10 mmol/l
  → Insulin Sliding Scale
- If patient has fever
  →Paracetamol 1g po TDS
- If patient has severe pain
  →Diclofenac 50mg po TDS
  → Morphine 5mg IV 6 hourly
- In case of autonomic dysregulation consider propranolol or clonidine

Monitoring

- Patella reflexes initially and then 6 hourly (Magnesium toxicity)
- Initial Lab: HIV rapid test, RBG, FBP, Creatinine, urine dipstick

↓

Wound care

- Alert surgeon for immediate and radical wound debridement
- Keep wounds open and clean

↓

Airway Management

- Early intubation if airway is compromised
- Consider early tracheotomy in case of laryngospasm or heavy secretion
- **Keep NPO for first 48 hours and do not place NG tube!**

↓

Prevent renal failure

- If urine is dark, reddish of inappropriate amount give 2 L NS immediately and prescribe 4L DNS or RL/24 hours

Spasms controlled within 4 hours?

Yes → **Continue Therapy**

No →

1. Stop the Mag bolus regime
2. Start infusion pump with 2g/h continuously IV
3. Reassess the patient
4. If still spasms, increase Diazepam dose (to maximum 40mg 4 hourly for adults)

Tetanus - Further management

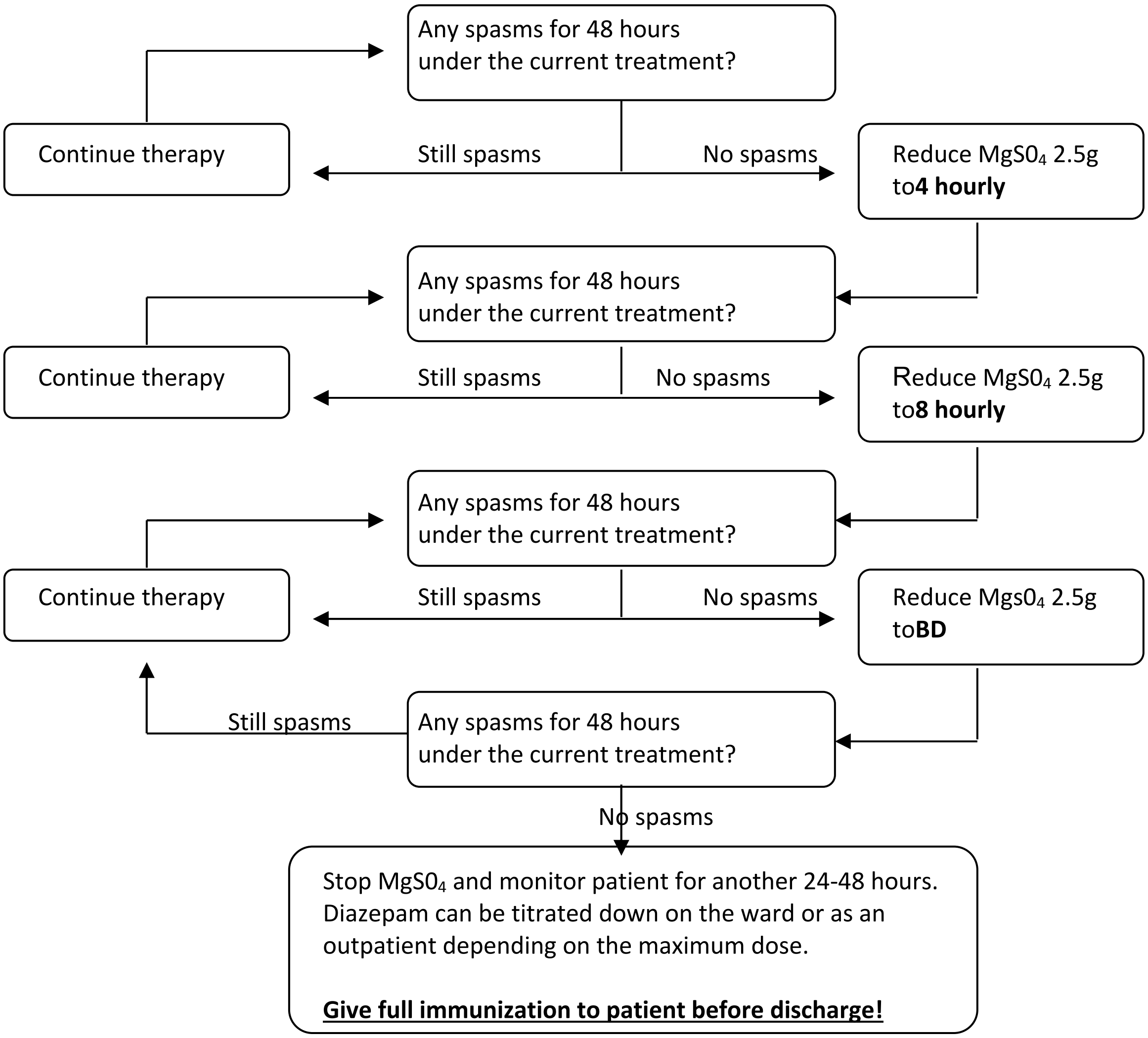

Ablett classification of Tetanus

- **Mild:** mild to moderate trismus, general spasticity, no respiratory embarrassment, no spasms, little or no dysphagia.
- **Moderate:** moderate trismus, marked rigidity, mild to moderate but short spasms, mild tachypnea with an ↑ RR>30, mild dysphagia.
- **Severe:** severe trismus, generalised spasticity, reflex prolonged spasms, ↑RR>40, apneic spells, severe dysphagia, tachycardia > 120.
- **Very severe:** grade III & violent autonomic disturbances involving the CVS. severe HTN & tachycardia alternating with relative hypotension & bradycardia, either of which may be present.
